# Effects of acute alcohol consumption on emotion recognition in social alcohol drinkers

**DOI:** 10.1177/0269881118822169

**Published:** 2019-02-05

**Authors:** Jasmine N Khouja, Angela S Attwood, Ian S Penton-Voak, Marcus R Munafò

**Affiliations:** 1 MRC Integrative Epidemiology Unit, University of Bristol, Bristol, UK; 2 School of Psychological Science, University of Bristol, Bristol, UK; 3 UK Centre for Tobacco and Alcohol Studies, University of Bristol, Bristol, UK

**Keywords:** Alcohol, alcohol-related aggression, emotion recognition, anger

## Abstract

**Background:**

Research suggests that acute alcohol consumption alters recognition of emotional expressions. Extending this work, we investigated the effects of alcohol on recognition of six primary expressions of emotion.

**Methods:**

We conducted two studies using a 2 × 6 experimental design with a between-subjects factor of drink (alcohol, placebo) and a within-subjects factor of emotion (anger, disgust, sadness, surprise, happiness, fear). Study one (*n* = 110) was followed by a direct replication study (*n* = 192). Participants completed a six alternative forced choice emotion recognition task following consumption of 0.4 g/kg alcohol or placebo. Dependent variables were recognition accuracy (i.e. hits) and false alarms.

**Results:**

There was no clear evidence of differences in recognition accuracy between groups (*ps* > .58). In study one, there were more false alarms for anger in the alcohol compared to placebo group (*n* = 52 and 56, respectively; *t*(94.6) = 2.26, *p* = .024, *d* = .44) and fewer false alarms for happiness (*t*(106) = –2.42, *p* = .017, *d* = –.47). However, no clear evidence for these effects was found in study two (alcohol group *n* = 96, placebo group *n* = 93, *ps* > .22). When the data were combined we observed weak evidence of an effect of alcohol on false alarms of anger (*t*(295) = 2.25, *p* = .025, *d* = .26).

**Conclusions:**

These studies find weak support for biased anger perception following acute alcohol consumption in social consumers, which could have implications for alcohol-related aggression. Future research should investigate the robustness of this effect, particularly in individuals high in trait aggression.

## Introduction

Facial emotional expressions are an important component of non-verbal communication, and deficits in the ability to recognise emotions are associated with impaired social function (Blair, 2003). Deficits in facial emotion recognition are common in several psychiatric disorders that are associated with poor social function, including autism (Uljarevic and Hamilton, 2013), schizophrenia (Barkhof et al., 2015) and depression (Bourke et al., 2010). Individuals with alcohol use disorder have also been shown to have deficits in facial emotion recognition, particularly for anger and disgust (Bora and Zorlu, 2017), and deficits prior to treatment are associated with less successful treatment outcomes (Rupp et al., 2017). In the wider population, poorer emotion recognition is associated with higher levels of aggression (Taylor and Jose, 2014) and anti-social behaviour (Marsh and Blair, 2008). A large body of evidence links alcohol consumption with aggressive behaviour, and reviews of experimental research suggest that this association is causal (Exum, 2006; Heinz et al., 2011). Given that ineffective emotional processing may increase the likelihood of aggression, it follows that alcohol might influence social interactions, including aggressive exchanges, by altering emotional face processing.

A number of studies report evidence of changes in emotional face processing following acute alcohol consumption, although the pattern of results is inconsistent across studies. Craig et al. (2009) and Kamboj et al. (2013) found acute alcohol consumption increased the perception of neutrality and the threshold for recognising sad facial expressions. Of note, sadness was not the only emotion included in these analyses. Craig and colleagues found no effect of acute alcohol consumption on thresholds for recognising happiness or anger (Craig et al., 2009), and although there was a tendency to show increased bias towards neutral faces when processing primary emotion expressions (happy, sad, anger, disgust and fear) further exploration by Kamboj and colleagues indicated this was limited to perception of sadness at lower doses (0.4 g/kg) and not higher doses (0.8 g/kg) of alcohol (Kamboj et al., 2013). Kano et al. (2003) found that higher alcohol doses (0.56 g/kg) worsened discrimination of happy faces compared to lower alcohol doses (0.28 g/kg), but no effect was found for discrimination of anger, sadness or surprise. Additionally, alcohol consumption has been shown to increase the accuracy of disgust and contempt recognition (Felisberti and Terry, 2015), and increase the perception of anger in ambiguous (i.e. anger-disgust) facial morphs (Attwood et al., 2009a). However, Felisberti and Terry (2015) did not find an effect for anger, fear, happiness or sadness, and perception of anger was only increased in the ambiguous angry-disgust facial morph (not the happy-angry facial morph) and only when the target stimulus was male (Attwood et al., 2009a).

Although differences in tasks and the emotional expressions used across studies makes direct comparisons difficult, inconsistencies between these findings suggest that further evidence is needed before reliable conclusions can be drawn about the effect of alcohol consumption on emotion recognition. Few previous studies presented all six primary emotional expressions (Felisberti and Terry, 2015; Kamboj et al., 2013), and therefore we cannot ascertain whether the effects of alcohol are specific to the presented emotions or represent a global effect on emotional face processing. Additionally, only one of the previous studies described investigated the effect using composite stimuli generated from multiple individuals (Craig et al., 2009) that reduce idiosyncratic differences between individuals to generate ‘prototypical’ facial expressions. None of the studies described explored all six primary emotions using composite images. Alcohol dosage also varied between studies. Higher doses of 0.8 g/kg can induce relatively widespread effects on pre-frontal functioning involved in attentional and motor control which can impact on general task performance whereas lower doses (0.4 g/kg) induce less widespread effects (Nikolaou et al., 2013). Therefore, lower doses of alcohol are preferable in order to induce changes in emotional processing whilst limiting confounding from impairments in attention and motor control.

To address the gap in the literature, we used a six alternative forced choice task (6AFC), which presents six primary emotions (i.e. anger, disgust, sadness, surprise, happiness, fear). Across two studies, we explored the effect of acute alcohol consumption (0.4 g/kg) on emotional face processing using the 6AFC task. The primary outcome variables were recognition accuracy (correct emotion identification) and false alarms (erroneous identification of absent emotions). Study two was a direct replication of study one, conducted to assess the robustness of our initial findings and further explore the effects on anger and happiness with an additional task. We combined both data sets to examine comparisons with increased power. Based on previous findings, we hypothesised that alcohol consumption would result in lower recognition accuracy (i.e. fewer hits) for sadness and more false alarms of negative emotions, particularly anger. In study two, we built upon the work of Attwood et al. (2009a) by including a newly developed two alternative forced choice task (2AFC) in which the dependent variable was the mean recognition balance point when attributing anger or happiness to emotional face morphs (using an angry-happy morph with varying intensities of anger and happiness displayed in each image). We hypothesised that there would be lower thresholds for identifying anger (i.e. an anger perception bias) following acute alcohol consumption compared to placebo.

## Study one

### Methods

#### Participants

Social alcohol consumers aged 18–40 years who declared themselves to be in good physical and psychological health were recruited from the University of Bristol (staff and students) and the general population by means of poster adverts, word of mouth and mailing lists. Participants were recruited if they consumed 10–50 alcoholic units (one unit equating to 10 ml of pure alcohol) per week if male or 5–35 alcoholic units per week if female to ensure alcohol was not a novel substance to the participant and to exclude potentially undiagnosed alcohol dependent individuals. Exclusion criteria included self-reported illicit substance (except cannabis) or psychiatric medication use to avoid confounding effects, self-reported direct family history of alcoholism (parent and/or sibling) for ethical reasons, alcohol consumption within 24 h of the study session (self-reported and breath alcohol concentration tested), and body weight of less than 50 kg if female or 60 kg if male (measured during the test session) for alcohol dosing purposes. Participants gave written informed consent prior to participating. Participants received £5 reimbursement upon completion of the study. Ethics approval was obtained from the University of Bristol’s Faculty of Science Human Research Ethics Committee (reference: 2609133923).

#### Design

We used a double-blind placebo-controlled experimental 2 × 6 mixed design with a between-subjects factor of drink (0.4 g/kg alcohol, placebo) and a within-subjects factor of emotion (anger, disgust, sadness, surprise, happiness, fear). Participants were allocated to the alcohol and placebo groups (which were stratified by sex) in order of attendance via a randomisation sheet generated using an online random number generator (www.randomizer.org).

#### Drink

Drinks were mixed based upon the participant’s weight (kg) and condition allocation. The alcoholic drink contained 0.4 g/kg with one part vodka to three parts tonic water, sufficient for achieving participant intoxication without expecting substantial motor deficits that may interfere with responding. Differences in emotion processing following alcohol compared to placebo have previously been seen with this dosage (Attwood et al., 2009a; Craig et al., 2009). Placebo drinks were matched by volume to the drinks in the alcohol condition but contained tonic water without vodka. Participants weighing over 90 kg received the same drink as a 90 kg participant. Drinks were chilled and flavoured with lime cordial (40 mL). The inside rim of the glass was sprayed twice with a vodka mist.

#### Measures and materials

Composite images (digital averages made from 12 individual exemplar faces) of each of six emotions (anger, disgust, sadness, surprise, happiness, fear) were used in the 6AFC task. The images were created using photographs of 12 young adult males posing expressions of happiness, sadness, anger, disgust, surprise and fear. The photographs were taken in a booth painted Munsel N5 grey which was illuminated with three Verivide F20 T12/D65 daylight simulation bulbs in high-frequency fixtures (Verivide, UK), which reduced the effects of flicker. For each emotion, a continuum of images was created in which intensity of displayed emotion increased from a prototypical ‘ambiguous’ face (emotionally ambiguous face created from composite images of the six emotions) to an unambiguous emotion. Experimental evidence indicates that such a prototype is likely to be a better approximation of the centre of emotional ‘face space’ than a neutral face (Skinner and Benton, 2010). Using standard computer graphic techniques (Tiddeman et al., 2001), 15-image morph sequences were created for each emotion; sequences ran from ‘ambiguous’ (5% along a linear continuum from ‘prototypical emotional ambiguity’ to ‘full intensity’ target emotion to ensure each face had some emotional signal) to ‘emotional’ (the ‘full intensity’ target emotion 100% along the continuum). Images were created using methods described in Bamford et al. (2015).

Questionnaire measures included the Alcohol Urges Questionnaire (AUQ; Bohn et al., 1995), the original Positive and Negative Affect Schedule (PANAS; Watson et al., 1988), the full Biphasic Alcohol Effects Scale (BAES; Martin et al., 1993) and the full self-report version of the Alcohol Use Disorders Identification Test (AUDIT; Saunders et al., 1993). AUDIT scores indicate lower risk (0–7), increasing risk (8–15), higher risk (16–19) or possible dependence (20+). The AUQ and BAES were used to indicate subjective changes which could indicate sufficient alcohol absorption to detect effects. The PANAS questionnaire was included to explore whether any effects on emotion recognition may be as a result of changes in own mood. AUDIT scores were used to indicate problematic drinking.

#### Procedure

Participants attended one test session at the University of Bristol. Upon arrival, participants were encouraged to re-read the information sheet before providing written informed consent. Participants were screened to confirm eligibility, which consisted of body weight measurement, an alcohol breath test (Draeger AlcoDigital 3000 Breathalyzer), carbon monoxide test (Bedfont PiCO+ Smokerlyzer) and verbal screening of the inclusion and exclusion criteria. Participants who failed to meet these criteria were excluded without reimbursement at this point.

Eligible participants completed baseline questionnaire measures (AUDIT, PANAS, BAES and AUQ) while a research collaborator delivered the drink and sealed envelope containing condition allocation information to the test room. A 10 min period was allowed for beverage consumption, followed by a 10 min absorption period, during which participants sat quietly in the test room. Participants then completed the 6AFC task. Verbal instructions were delivered to the participant and they were given the opportunity to ask questions. Written instructions were displayed on screen at the beginning of the task informing the participant that they would have to judge the emotion displayed briefly on a face and respond by clicking the emotion label as quickly as possible. Over 180 trials and approximately 12 min, the six 15-image sequences were displayed twice at random. Trials began with the presentation of a fixation point (either 1500 ms or 2500 ms at random), followed by the presentation of one face (150 ms). Next, a backward mask of visual noise (to prevent processing of afterimages) was displayed (250 ms) and then a six-choice array of descriptors (anger, disgust, sadness, surprise, happiness, fear) were displayed in a circular arrangement. Participants selected the emotion in the face presented from an array using the computer mouse. The emotion array was displayed for 10,000 ms, or until the participant selected an emotion. Primary outcomes were the number of correct emotion identifications (i.e. hits) and the number of incorrect identifications (i.e. false alarms). Questionnaire measures (PANAS, BAES and AUQ) were completed again following the computer task. A verbal and written debriefing and £5 reimbursement were given at the end of the session. Participants in the alcohol condition signed post-session safety forms confirming they understood they had consumed alcohol and should avoid activities considered dangerous under the influence of alcohol. These participants were given the option of staying behind in a quiet room until the effects of the alcohol wore off and were offered a paid taxi home.

#### Statistical analysis

The findings from Craig et al. (2009) indicated a large effect size of *d* = 1.0 for the difference between alcohol and placebo on sadness recognition (*M* = 0.14, SD = 0.02; *M* = 0.12, SD = 0.02, respectively). As this is likely to be an inflated effect size (Button et al., 2013), we used a more conservative, medium effect size estimate of *d* = 0.5 (equivalent to a between group difference of 1.68 hits for sadness) to calculate the sample size for the current study. We calculated 105 participants were needed to achieve 95% power to detect a main effect of alcohol at an alpha level of 5%. In order to balance groups for gender, we recruited 110 participants. This sample size would provide 80% power to detect a medium interaction (drink × emotion) effect size equivalent to *f* = 0.25 at an alpha level of 5%.

Statistical analyses were conducted using IBM SPSS Statistics (Version 21). Outliers (total hit scores more than 1.5 times the interquartile range above the upper or below the lower quartile) were identified using boxplots and were removed from further analysis (*ns* reported in the results). No substantial deviations from normality were observed based on skewness and kurtosis statistics. Greenhouse–Geisser statistics are reported where Mauchly’s Test of Sphericity was *p* < .05. Hits by emotion were assessed in a 2 drink (alcohol, placebo) × 6 emotion (anger, disgust, sadness, surprise, happiness, fear) analysis of variance (ANOVA). Post hoc independent samples t-tests were conducted. False alarms for each emotional expression were analysed using a series of independent t-tests.

The data that form the basis of the results presented here are available from the data.bris Research Data Repository (http://data.bris.ac.uk/data/), DOI: 10.5523/bris.1bc7x6ninwtee2jhlvf7fp0r0g.

### Results

#### Participant characteristics

Data were collected from 110 participants (50% male). Data from one participant were removed from all analyses due to a randomisation error. One outlier was identified from the total hits data and excluded from further analysis (including data from this participant resulted in no substantial changes to the results reported). Participants included in the analyses (*n* = 108; 50% male) were aged 18–39 years (*M* = 21; SD = 4) and weighed 50–121 kg (*M* = 69; SD = 13). AUDIT scores ranged from 4 to 19 (*M* = 11; SD = 4). Participants were older on average in the placebo group (*M* = 22) than the alcohol group (*M* = 21) and weighed less on average in the placebo group (*M* = 68 kg) than the alcohol group (*M* = 70 kg). AUDIT scores were slightly lower on average in the placebo group (*M* = 10) than the alcohol group (*M* = 11). In the placebo group, 9% of participants had CO readings of 10 parts per million (ppm) or above indicating they were active smokers compared to 10% in the alcohol group. On completion of the study, fewer participants who received a placebo drink believed they had received an alcoholic drink (50%) compared to those in the alcohol condition (96%).

#### Hits by emotion

**Figure 1.**
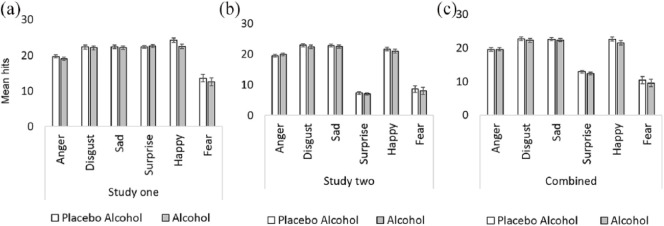
Mean (± SE) recognition accuracy (hits) for all six primary emotional expressions in study one (a), study two (b) and the combined data (c). Note: SE: standard error. Hits refer to the average number of stimuli in which the emotions were accurately recognized.

There was a main effect of emotion (*F*[3.1, 329] = 83.1, *p* < .001, *η_p_*^2^ = .44) with fear having the fewest hits and the most hits for happiness, but no clear evidence of a main effect of drink, (*F*[1, 106] = 1.44, *p* = .23, *η_p_*^2^ = .013), or an emotion × drink interaction (*F*[3.1, 329] = 0.67, *p* = .58, *η_p_*^2^ = .006) (Figure 1).

#### False alarms by emotion

Independent samples *t*-tests indicated evidence of more anger false alarms (*t*[94.6] = 2.26, *p* = .024, *d* = .44) in the alcohol compared to the placebo condition (*M* = 4.6 SD = 4.1; *M* = 3.0, SD = 3.1, respectively) and fewer happy false alarms (*t*[10]) = –2.42, *p* = .017, *d* = –.47) in the alcohol condition compared to the placebo condition (*M* = 9.1, SD = 7.6; *M* = 13.1, SD = 9.4, respectively). There was no clear evidence of drink group differences on any other emotion (*p*s > .17; see Table 1). Adjusting for age and weight did not alter these results substantially.

**Table 1.** Mean differences between the alcohol and placebo groups including 95% confidence intervals, *p*-values and effect sizes for the misattribution (false alarms) of each six primary emotional expressions in study one, study two and the data from both studies combined.

Study	Mean difference	95% CI	*p*-value	Effect size (*d*)
Anger				
1	1.60	0.19, 3.00	.024	.44
2	0.85	−0.51, 2.21	.22	.18
Combined	1.14	0.14, 2.14	.025	.26
Disgust				
1	0.79	−0.97, 2.54	.38	.17
2	0.95	−1.57, 3.48	.46	.11
Combined	1.00	−0.78, 2.79	.27	.13
Sadness				
1	1.84	−0.78, 4.45	.17	.27
2	0.13	−2.26, 2.52	.91	.02
Combined	0.83	−0.99, 2.64	.37	.10
Surprise				
**1**	2.23	−1.38, 5.85	.22	.23
2	0.20	−2.17, 2.57	.87	.02
Combined	1.08	−1.01, 3.18	.31	.12
Happiness				
1	−3.97	−7.23, −0.71	.017	−.47
2	−0.77	−4.24, 2.71	.67	−.06
Combined	−1.85	−4.38, 0.68	.15	−.17
Fear				
1	1.11	−1.97, 4.19	.48	.14
2	0.46	−2.84, 3.77	.78	.04
Combined	0.78	−1.62, 3.18	.52	.07

95% CI: 95% confidence interval.

#### Sensitivity analysis

Six individuals weighed more than 90 kg and therefore received a lower dose than 0.4 g/kg of body weight, so we conducted sensitivity analyses whereby individuals weighing over 90 kg were excluded. We found similar results with slightly stronger evidence of the effects (results not shown).

#### Questionnaire data

Results of the questionnaires are displayed in Table 2. There was no clear evidence of a main effect of time (*p* = .37) or drink (*p* = .59) for AUQ scores indicating the placebo was effective. However, there was weak evidence of a small drink × time interaction effect (*F*[1, 106] = 5.68, *p* = .019, *η_p_*^2^ = .051), with decreased alcohol urges in the placebo group and increased urges in the alcohol group across the session.

**Table 2.** Means and standard deviations pre- and post-drink questionnaire scores for study one and two for the total sample, placebo group and alcohol group.

Questionnaire	Sample	Study one (*n* = 108)				Study two (*n* = 189)			
		Pre-drink		Post-drink		Pre-drink		Post-drink	
		M	SD	M	SD	M	SD	M	SD
AUQ	Total	13.4	8.9	14.2	11.9	29.4	6.5	27.7	7.5
	Placebo	14.1	7.9	12.6	10.7	13.0	10.0	12.3	10.8
	Alcohol	12.7	9.9	15.9	12.9	13.5	9.6	15.9	12.8
PANAS positive	Total	28.5	7.1	25.5	8.0	13.5	9.6	15.9	12.8
	Placebo	29.5	7.0	25.6	7.5	29.8	6.7	27.5	7.1
	Alcohol	27.3	7.2	25.4	8.6	29.1	6.4	27.9	8.0
PANAS negative	Total	12.5	2.7	11.9	2.3	12.7	3.4	12.5	3.6
	Placebo	12.6	2.7	11.7	2.1	12.5	3.1	12.4	3.5
	Alcohol	12.5	2.7	12.1	2.4	12.9	3.7	12.6	3.7
BAES stimulation	Total	36.2	11.8	31.3	12.9	36.5	9.5	34.4	10.8
	Placebo	37.5	12.2	29.2	12.5	36.6	8.7	32.9	10.2
	Alcohol	34.9	11.3	33.5	13.2	36.4	10.3	35.8	11.3
BAES sedation	Total	15.7	10.4	24.2	13.2	16.5	11.0	24.9	13.0
	Placebo	14.6	10.7	22.6	12.9	16.8	11.2	22.2	12.8
	Alcohol	16.9	10.1	26.0	13.4	16.1	10.8	27.6	12.8

AUQ: Alcohol Urges Questionnaire; PANAS: Positive and Negative Affect Schedule; BAES: Biphasic Alcohol Effects Scale; SD: standard deviation.

Main effects of time were observed for positive (*F*[1, 106] = 21.1, *p* < .001, *η_p_*^2^ = .17) and negative affect (*F*[1, 106] = 5.58, *p* = .020, *η_p_*^2^ = .05), with both decreasing across the session. There was no clear evidence of main effects of drink or of drink × time interactions on positive or negative affect (*ps* > .11) indicating the effects of alcohol on emotion processing were not due to changes in participant mood.

There was strong evidence of main effects of time for BAES stimulation scores (*F*[1, 106] = 20.3, *p* < .001, *η_p_*^2^ = .16) which decreased across the session, and sedation scores (*F*[1, 106] = 50.8, *p* < .001, *η_p_*^2^ = .32), which increased across the session. There was no clear evidence of main effects of drink for stimulation (*p* = .67) or sedation (*p* = .15) scores. Strong evidence of a time x drink interaction was observed for stimulation scores (*F*[1, 106] = 10.3, *p* = .002, *η_p_*^2^ = .089) but not for sedation scores (*p* = .64). Stimulation scores decreased across the session in both groups but more so in the placebo condition. Changes in stimulation scores between groups across time indicate the placebo was effective and that the alcohol dose was sufficient to see effects on subjective measures.

To explore whether these results were robust, we ran a replication study, with a larger sample size to increase the power to detect an effect. We calculated the sample size for this study using the results for anger false alarms from study one. These results were used rather than the results for happiness false alarms because we wanted to have enough power to detect the weaker of our two strongest findings.

## Study two

### Methods

This study was pre-registered on the Open Science Framework (doi: 10.17605/OSF.IO/6I3JG). Except where noted, the methods for study two were identical to study one.

#### Measures

A 2AFC task was added to the procedure alongside the 6AFC task. Building on the work of Attwood et al. (2009a), this revised 2AFC task included new composite stimuli designed to reduce idiosyncratic differences by merging the images of multiple actors into one face displaying the ‘average’ of each emotion. One 15-image sequence displayed 100% happiness at one end of the continuum and morphed gradually image by image into 100% anger. Each image between the two full intensity images contained a proportion of both emotions (i.e. 90% happiness contained 10% anger).

#### Procedures

Directly following the 6AFC task, verbal and written computerised instructions were given to the participant and the 2AFC task was completed. Participants were instructed they would judge the emotion on a face briefly displayed by pressing ‘c’ or ‘m’ on the keyboard as quickly as they could. The 2AFC task used the same presentation settings as the 6AFC task. Each image was presented three times (45 total trials) taking approximately 3 min. Participants identified whether the emotion displayed was anger or happiness by pressing the ‘m’ and ‘c’ keys on a QWERTY keyboard respectively. The primary outcome was an estimate of the point on the continuum at which the participant was equally likely to respond ‘happy’ or ‘angry’ (the ‘balance point’ or ‘threshold’). The balance point was estimated by calculating the number of ‘happy’ responses proportionate to the number of trials.

#### Statistical analysis

As stated in our pre-registered online protocol, a sample size calculation was based on an effect size (*d* = .41) obtained from study one (difference between false alarms to anger in alcohol and placebo conditions). Based on these data, a sample size of 190 was required to achieve 80% power at an alpha level of 5%. This sample size was calculated before the randomisation error described above was identified. The correct effect size observed in study one was greater (*d* = .44), therefore a sample of 190 would provide 85% power.

Statistical analyses were conducted using IBM SPSS Statistics (Version 21). Outliers (total hit scores more than 1.5 times the interquartile range above the upper or below the lower quartile) were identified using boxplots and were removed from further analysis (*ns* reported in the results). No substantial deviations from normality were observed based on skewness and kurtosis statistics.

The analysis method (ANOVA) which we stated in the pre-registered protocol would be used to analyse false alarms in the 6AFC was later considered to be inappropriate. False alarms are only meaningful when considered at an emotion specific level (i.e. are inverse of hits at the global level), and consequently are not independent (i.e. an increase in false alarm rate to one emotion will impact on false alarm rates across other emotions). Therefore, emotion specific false alarm rates were analysed using six independent-samples *t*-tests. For the 2AFC data, mean balance points were analysed in a between-groups independent *t*-test to determine whether there was an attribution bias towards the recognition of either happiness or anger following drink consumption.

The data that form the basis of the results presented are available from the data.bris Research Data Repository (http://data.bris.ac.uk/data/), DOI: 10.5523/bris.15guozp49qaxl2s1giypxb9rb9.

### Results

#### Participant characteristics

Data from 192 participants were collected (50% male). Results are reported excluding three outliers (*n* = 189; 49% male). Inclusion of these data did not cause any substantial changes to the results reported. Participants were aged 18–39 years *(M* = 22; SD = 4) and weighed 51–120 kg (*M* = 69; SD = 12). AUDIT scores ranged from 3 to 24 (*M* = 10; SD = 4). Participants were slightly older on average in the placebo group (*M* = 23 years) than the alcohol group (*M* = 22 years). Participants also weighed less in the placebo group (*M* = 69 kg) than the alcohol group (*M* = 70 kg) but average AUDIT scores were the same on average in both groups. In the placebo group, 10% of participants had CO readings of 10 ppm or above indicating they were active smokers compared to 14% in the alcohol group. The mean breath alcohol concentration reached in the alcohol group was 17 µg/100 mL (SD = 5) and 0 µg/100 mL in the placebo group (*SD* = 0). A data collection error resulted in incomplete manipulation check data for 49 participants. Of those with manipulation check data (*n* = 141), 45% believed their placebo drink contained alcohol. This considerably differed from those in the alcohol condition where 90% believed their drink contained alcohol (*p* < .001).

#### Hits by emotion (6AFC task)

There was strong evidence of a main effect of emotion (*F*[3.98, 745] = 451, *p* < .001, *η_p_*^2^ = .71) with disgust having the most hits and surprise having the least hits, but not of a main effect of drink (*F*[1, 187] = 1, *p* = .32, *η_p_*^2^ = .05) or of an emotion × drink interaction (*F*[3.98, 742] = 0.35, *p* = .84, *η_p_*^2^ = .002) (Figure 1).

#### False alarms by emotion (6AFC task)

There was no clear evidence of differences between groups for false alarms on any of the emotions (*ps* > .22, see Table 1).

#### Recognition balance points (2AFC task)

There was no clear evidence of a mean balance point difference in anger recognition between the alcohol and placebo group (*t* [187] = 0.43, *p* = .67, *d* = .061).

#### Sensitivity analysis

Eleven individuals weighed more than 90 kg and therefore received a lower dose than 0.4 g/kg of body weight, so we conducted sensitivity analyses whereby individuals weighing over 90 kg were excluded. We found similar results with slightly weaker evidence of the effects (results not shown), however, there was no change to the interpretation of the results.

#### Questionnaire data

Descriptive data for the questionnaire measures can be found in Table 2. There was no clear evidence for a main effect of time or drink (*ps* > .16) on AUQ scores. There was some evidence of a drink × time interaction (*F*[1, 184] = 5.7, *p* = .018, *η_p_*^2^ = .03) with decreased alcohol urges in the placebo group and increased urges in the alcohol group across the session indicating the placebo was effective.

There was strong evidence of a main effect of time (*F*[1, 185] = 21.7, *p* < .001, *η_p_*^2^ = .11) for positive PANAS scores, with increased scores across the session. There was no clear evidence of a main effect of time for negative scores or a main effect of drink for positive or negative scores (*ps* > .37) or of time × drink interactions for positive or negative scores (*ps* > .17) indicating the effects of alcohol on emotion processing were not due to changes in participant mood.

Strong evidence for main effects of time was found for BAES stimulation scores (*F*[1, 185] = 11.3, *p* = .001, *η_p_*^2^ = .058) and sedation scores (*F*[1, 185] = 121.6, *p* < .001, *η_p_*^2^ = .4). Stimulation scores decreased and sedation scores increased across the session. No clear evidence for main effects of drink was found for stimulation or sedation (*ps* > .14). There were time × drink interactions for stimulation (*F*[1, 185] = 6.18, *p* = .014, *η_p_*^2^ = .033) and sedation (*F*[1, 185] = 15.9, *p* < .001, *η_p_*^2^ = .079) scores indicating the placebo was effective and that the alcohol dose was sufficient to see effects. There were lower stimulation scores in both the placebo and alcohol group post-drink consumption whereas sedation scores increased across the session in both conditions.

## Combined data

Main analyses were repeated for the data sets of study one and two combined. A covariate of study (1, 2) was included in an ANCOVA of hits by emotion. The combined results were similar to the separate results of study one and two in terms of mean hits by emotion (Figure 1). However, there was evidence of an emotion × study interaction (*F*[3.81, 1120] = 123, *p* < .001, *η_p_*^2^ = .3). The combined results for false alarms are shown in Table 1. There was no clear evidence of a difference between groups for false alarms of happiness (*t*[295] = –1.44, *p* = .15, *d* = −.17). However, there was weak evidence of a between groups difference in anger false alarms where more false alarms occurred in the alcohol than the placebo group (*t*[295] = 2.25, *p* = .025, *d* = .26). Adjusting for age and weight did not alter these results substantially.

## Discussion

Using novel methods including composite images of six emotions in varying intensities, these results provided some evidence that acute alcohol consumption affects emotional face processing. Alcohol increased the number of false alarms to angry and decreased false alarms to happy facial expressions in study one. While these effects did not directly replicate in study two, the same pattern was observed for angry faces, and the effect remained when the data from the two studies were combined, suggesting alcohol consumption has an emotion-specific rather than global effect, affecting only anger processing. However, the statistical evidence from the combined analysis remained relatively weak. Assuming our experimental tasks validly assess emotion processing in the real world, and that small changes in perception lead to small changes in behaviour, this indicates that any true effects on behaviour are likely to be small. There was no clear evidence of an effect of acute alcohol consumption on recognition accuracy (i.e. hits) and no difference in mean recognition balance points (2AFC task) between groups. These results indicate that any genuine effects of alcohol on emotion processing are likely to be smaller than indicated by studies that use smaller sample sizes than we used here.

The lack of alcohol effect on recognition accuracy is in contrast to previous research where acute alcohol consumption led to decreased recognition of sadness (Craig et al., 2009; Kamboj et al., 2013) and increased accuracy of disgust recognition (Felisberti and Terry, 2015). Instead alcohol only affected false alarms, which is indicative of ‘bias’. This parallels emotion recognition of ambiguous images in depression, where negative bias in the interpretation of neutral faces occurs without deficits in emotion recognition (Maniglio et al., 2014). This may be partly due to a dose effect, as our study used a lower dose (Felisberti and Terry, 2015) (0.6 g/kg).

In line with our finding that false alarms for anger increase after alcohol consumption, Attwood et al. (2009a) observed a bias towards perceiving anger (in anger-disgust facial morphs) following alcohol consumption using a 2AFC task. Consistent with our 2AFC findings in study two, this study did not find evidence of an alcohol effect when the task presented facial morphs comprising a mixture of anger and a positive emotion (i.e. happiness). As there is a recognition advantage for happy faces (Calvo and Beltran, 2013), anger biases after acute alcohol consumption may only be observed when angry faces are morphed with negative emotional expressions. Furthermore, this effect may impact on consequent behaviour. Wilkowski and Meier (2010) found that angry faces are more likely to be approached rather than avoided. This adaptive response to protect one’s resources (Sell et al., 2009) or social relationships (Fischer and Roseman, 2007; Gottman and Krokoff, 1989) could explain increased aggression following acute alcohol consumption; intoxicated individuals aggressively approach people who are seemingly expressing anger as a defensive strategy.

Alcohol-related aggression is greater in individuals with higher trait aggression (Giancola et al., 2002; Tremblay et al., 2007, 2008). This group also show greater emotional processing deficits in sober states (Hall, 2006). Therefore, the effects of alcohol on emotion processing, particularly anger biases, may be greater in high trait aggressive individuals, which could consequently increase the likelihood of alcohol-related aggression in these individuals. In the current study, we recruited an unselected sample of social alcohol consumers, and therefore it is unsurprising that small effect sizes were obtained. Research is needed to investigate whether alcohol effects on emotional processing are greater in high trait aggressive individuals. Using a within-participant design to compare individuals high and low in trait aggression, across multiple sessions where they receive either alcohol or placebo, could address this question. This in turn may explain some of the individual variability in the alcohol-aggression relationship. Further, our findings highlight the possibility that previous studies may have been underpowered to detect any genuine effects of alcohol on emotion recognition and underline the need for large sample sizes in future research which will reduce the likelihood of both type I and type II errors (Christley, 2010).

Although the evidence we have presented is supported by some previous studies, there is a degree of inconsistency in the literature regarding the nature of the effect alcohol consumption on emotion recognition. We found no evidence to suggest that alcohol affected the perception of sadness or disgust as found in previous studies (Craig et al., 2009; Felisberti and Terry, 2015; Kamboj et al., 2013). Additionally, despite having included anger stimuli, no effect of alcohol consumption on anger perception was found in some previous studies (Craig et al., 2009; Felisberti and Terry, 2015; Kano et al., 2003). A possible explanation for these inconsistencies could be methodological differences between the studies.

A key difference between the methodology of this study and most previous studies in the field is that an emotionally ambiguous face was created from composite images of the six emotions. Psychophysical adaptation studies suggest that this is arguably a more accurate representation of the centre of the emotion ‘face space’ than a neutral face (commonly used in the literature) and therefore should decrease the likelihood that participants biases were due to the ambiguous face being closer on the emotional continuum to one emotion than another (Skinner and Benton, 2010). Merging identities into composite images reduced idiosyncratic differences in the emotions displayed. The use of composite images in this research, rather than non-composite images (i.e. a single identity) which have been used in previous research (Attwood et al., 2009a), supports the theory that emotion recognition is affected by alcohol consumption and that the effects are not due to idiosyncratic image differences. Further research is needed using composite images in 2AFC tasks in which negative emotions (disgust, sadness and fear) are morphed into anger to see whether there are global or specific negative emotion biases.

Strengths of this work include the large sample sizes in both studies, the increased power resulting from combining the studies and the use of a double-blind procedure. The novel use of composite images in a task displaying anger, happiness, disgust, sadness, fear and surprise is another strength as it indicates, for the first time, that when idiosyncratic differences are accounted for, changes in emotion recognition due to acute alcohol consumption are specific and not global. However, these results should be treated with caution as the studies are not without limitations. Our second study was powered to detect a moderate effect yet did not directly replicate study one and the effects that were detected were small and unlikely to be clinically or socially important. Additionally, higher alcohol doses than 0.4 g/kg could reveal a larger effect of alcohol on emotion recognition as more alcohol-related aggression is seen following higher doses (Exum, 2006). However, if the alcohol dose were to be increased, the placebo control would also need to be improved; at 0.4 g/kg of alcohol versus 0.0 g/kg placebo, participants were substantially more likely to believe they received alcohol and this difference may be greater when using higher doses. Studies that deliver intoxicating doses of alcohol often suffer from this limitation. A further issue with using higher doses would be the increased motor impairments which would occur in the alcohol group. The inclusion of a psychomotor task could allow the estimation of the magnitude of these effects, which could in principle then be adjusted for in analyses of the effects of alcohol on emotion processing. Although alcohol dependent individuals should not have been included in the sample, it is also possible that some individuals were alcohol dependent because using 50 units or more as a cut off for weekly alcohol consumption is not sufficient to exclude those who may meet the criteria for a diagnosis of alcohol dependence. Another limitation is that static images were used in the studies rather than dynamic stimuli; static images may not be as realistic as dynamic videos of intensifying emotion such as those used by Kamboj et al. (2013).

In the future, replicating these methods using a dose ranging study similar to the research of Attwood et al. (2009b) with doses higher than 0.4 g/kg could explore the impact of alcohol dose on emotion recognition. Future studies may also be enhanced with the use of composite dynamic stimuli which may be more realistic but not influenced by idiosyncratic differences.

## Conclusions

The effects found in this study of social drinkers are in line with some previous findings, providing some support for an increased bias towards anger during facial emotion recognition following alcohol consumption. However, to the extent that our experimental measures validly measure real world emotion processing, we can conclude that any effect on behaviour is likely to be small. Further exploring these effects in sub-populations, such as individuals with high trait aggression, may reveal larger effects which could help to understand the underlying mechanisms involved in alcohol-related aggression.
